# Real-Time Sensing of Upper Extremity Movement Diversity Using Kurtosis Implemented on a Smartwatch

**DOI:** 10.3390/s24165266

**Published:** 2024-08-14

**Authors:** Guillem Cornella-Barba, Shusuke Okita, Zheng Li, David J. Reinkensmeyer

**Affiliations:** 1 Department of Mechanical and Aerospace Engineering, University of California Irvine, Irvine, CA 92697, USA; dreinken@uci.edu; 2 Max Näder Center for Rehabilitation Technologies and Outcomes Research, Center for Bionic Medicine, Shirley Ryan AbilityLab, Chicago, IL 60611, USA; sokita@sralab.org; 3 School of Electronics, Electrical Engineering and Computer Science, Queen’s University Belfast, Belfast BT7 1NN, UK; imlizheng@gmail.com; 4 Department of Anatomy and Neurobiology, University of California Irvine, Irvine, CA 92697, USA

**Keywords:** wearable activity sensing, accelerometer, tilt sensing, rehabilitation, upper extremity movement diversity, kurtosis, rolling statistics

## Abstract

Wearable activity sensors typically count movement quantity, such as the number of steps taken or the number of upper extremity (UE) counts achieved. However, for some applications, such as neurologic rehabilitation, it may be of interest to quantify the quality of the movement experience (QOME), defined, for example, as how diverse or how complex movement epochs are. We previously found that individuals with UE impairment after stroke exhibited differences in their distributions of forearm postures across the day and that these differences could be quantified with kurtosis—an established statistical measure of the peakedness of distributions. In this paper, we describe further progress toward the goal of providing real-time feedback to try to help people learn to modulate their movement diversity. We first asked the following: to what extent do different movement activities induce different values of kurtosis? We recruited seven unimpaired individuals and evaluated a set of 12 therapeutic activities for their forearm postural diversity using kurtosis. We found that the different activities produced a wide range of kurtosis values, with conventional rehabilitation therapy exercises creating the most spread-out distribution and cup stacking the most peaked. Thus, asking people to attempt different activities can vary movement diversity, as measured with kurtosis. Next, since kurtosis is a computationally expensive calculation, we derived a novel recursive algorithm that enables the real-time calculation of kurtosis. We show that the algorithm reduces computation time by a factor of 200 compared to an optimized kurtosis calculation available in SciPy, across window sizes. Finally, we embedded the kurtosis algorithm on a commercial smartwatch and validated its accuracy using a robotic simulator that “wore” the smartwatch, emulating movement activities with known kurtosis. This work verifies that different movement tasks produce different values of kurtosis and provides a validated algorithm for the real-time calculation of kurtosis on a smartwatch. These are needed steps toward testing QOME-focused, wearable rehabilitation.

## 1. Introduction

For people without movement-related disabilities, wearable sensors have proven useful for promoting physical activity. For example, setting a daily step goal and then monitoring it with daily step count feedback increases walking activity, thereby improving difficult-to-change health outcomes such as blood pressure [1]. Unfortunately, the few research studies that have examined the use of wearable feedback to promote greater physical activity of either walking or upper extremity (UE) use after stroke have had limited success (see review [2]). We propose testing a different approach for neurologic movement rehabilitation: using goal setting and wearable feedback to encourage people to explore new (rather than just more of the same) movement patterns, thereby challenging their motor system [3].

We envision a home rehabilitation scenario in which a person with a neurologic impairment wears a simple, low-cost sensor on their wrist, hip, or ankle, and that sensor vibrates when they succeed in moving in new ways, i.e., in ways that are unusual and challenging for them. The sensor could also keep track of the total minutes per day they spend exploring. We hypothesize that this would motivate people to set a goal to seek new movement experiences. We also hypothesize that seeking a greater amount of movement diversity would help activate forms of neuroplasticity that improve motor control, reduce impairment, and help people become more active.

Note that the approach we are proposing is different than quantifying the quality of individual movements. Higher-quality, individual movements have been defined in different ways in the context of UE rehabilitation, including movements that are smoother [4], faster [5], that do not rely on compensatory patterns such as leaning with the trunk [6], and that do not rely on abnormal synergistic patterns of movement [7]. The approach we call Quality of Movement Experience (QOME) focuses on the statistical properties of extended periods of many movements throughout the day (or “movement epochs”), a concept related to previous work aimed at optimizing movement experiences and exploration in robotic therapy sessions [8,9,10].

One possible strategy for quantifying QOME would be to identify different physical activities when a person tries them, a well-studied strategy that relies on machine learning [11,12]. We believe this approach is suboptimal because accurate classification requires using relatively small sets of candidate activities, but, in real life, people can leverage a vast number of activities to promote physical activity and rehabilitation. We, therefore, propose to focus instead on more general statistical characteristics of daily UE and lower extremity (LE) movements that become more prominent as UE and LE impairment decrease. In this framework, the goal is to motivate people to move more frequently like people who have experienced incrementally better recovery.

In a previous study from our lab [13] (in press), we tested the feasibility of identifying such characteristics using raw wrist acceleration data obtained from 22 participants with a stroke. Table 2 from [13] presents the measures that most effectively distinguished impairment levels when subjects were grouped into severe, moderate, and mild impairment categories. From a group of 10 candidate statistics, we found that forearm postural diversity (quantified by the kurtosis of the tilt angle distribution) had the strongest power to distinguish severe and moderate impairment groups (Cohen’s D = 1.1, *p* = 0.028, indicating a strong effect size). In addition, in [13], when we plotted kurtosis versus impairment levels (quantified by the Upper Extremity Fugl-Meyer score [14]) for individuals across all impairment levels, we found that there was a significant correlation between kurtosis and impairment level (r = 0.374, *p* = 0.029). However, kurtosis could not distinguish between moderate and mild impairment, and instead, Sample Entropy of the tilt angle—a measure of movement complexity—was most effective (Cohen’s D = 0.99, *t*-test, *p* = 0.017). In this paper, we focus on quantifying movement diversity with kurtosis, and, thus, this work is most relevant to individuals with severe/moderate impairment after stroke.

**Figure 1:**
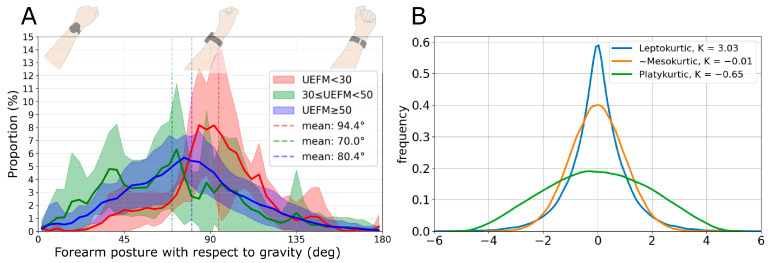
(**A**): The distribution of the device tilt angle and corresponding forearm postures with respect to gravity. The red, green, and blue lines represent the mean distribution for groups with varying impairment levels [13]. UEFM stands for the Upper Extremity Fugl-Meyer score [14], a test of arm impairment that varies from 0 (complete paralysis) to 66 (normal movement). (**B**): Different kurtosis values for three distributions: mesokurtic (similar to normal), leptokurtic (more peaked than normal), and platykurtic (more tailed than normal).

It makes sense that individuals with better UE recovery achieve a greater diversity of forearm postures throughout the day as they engage in a greater variety of physical activities and/or do the same activities but with improved movement quality, such as a better range of motion. Kurtosis is a well-established measure that quantifies the sharpness of the peak of a frequency distribution curve in comparison to the normal distribution (see Figure 1 [15]). This works well for quantifying diversity—if your arm stays in the same posture all day long (as can happen after a severe stroke), then the frequency distribution of forearm posture will be sharply peaked with high kurtosis, consistent with movement stagnation. More diverse movement experiences will have more flat and distributed postures, exhibiting low kurtosis.

The purpose of this experimental and mathematical study is to present further work toward the wearable sensing of movement diversity using kurtosis, focusing on three objectives. First, we consider the objective of determining how to intentionally increase diversity in daily life. Our ultimate goal is to develop an algorithm for a smartwatch that informs users when they are moving in more diverse ways (relative to their baseline) and/or tells them how many minutes they spend moving in new ways each day. The idea is that, when users receive feedback that their diversity has been high, they will be motivated to repeat these diversity-enhancing activities. However, the hypothesis that engaging in different activities can improve diversity, as detected with kurtosis, remains uncertain. To test this hypothesis, we evaluated a set of 12 activities (including a booklet of conventional rehabilitation therapy exercises, Cornhole, playing cards, etc.) for the values of kurtosis they produced.

Second, calculating kurtosis is computationally complex due to its reliance on calculating the fourth moment around the mean, and, to our knowledge, it has only been calculated offline. A critical aspect of the kurtosis computation is its sequential dependence, necessitating the prior calculation of both mean and standard deviation. Thus, a second objective was to develop a novel, recursive kurtosis algorithm to speed up computation time and reduce battery consumption, suitable for implementation on a smartwatch.

Third, a key problem in implementing wearable sensing algorithms is the difficulty in assessing ground truth. One can implement a kurtosis algorithm on a smartwatch, but verifying its correctness remains a challenge. Here, we used a custom-built robotic device to create movement patterns with known kurtosis values from predefined trajectories. This allowed us to assess the hypothesis that the novel kurtosis algorithm, as implemented on a smartwatch, could accurately calculate kurtosis in real time. Thus, we made progress toward the objective of verifying real-time kurtosis calculations.

In summary, the main aim of this work is to make progress toward the wearable sensing of movement diversity using kurtosis, a need in rehabilitation research to enable testing of the hypothesis that Quality of Movement Experience (QOME) feedback could improve recovery. As we will show, we (1) verified that different movement tasks have different kurtosis values; (2) derived a novel, recursive algorithm that dramatically improves the computation speed of kurtosis; and (3) used a robot arm to demonstrate that the algorithm accurately computes kurtosis in real time.

## 2. Materials and Methods

### 2.1. Experiment 1: Do Different Therapeutic Activities Generate Different Values of Kurtosis?

We recruited seven unimpaired individuals (aged 20–25, all male) and evaluated a set of 12 therapeutic activities for forearm postural diversity. The study was deemed exempt from IRB review using the UCI IRB exempt self-determination tool under Category 3i: Behavioral Interventions.

The activities included the following: (a) a set of conventional rehabilitation therapy exercises for the hand and arm that were described in a booklet (used in a previous clinical trial of home-based stroke rehabilitation [16]), (b) ping-pong, (c, d) the card games Speed and Klondike, (e) American Sign Language, (f) Tai-chi, (g) Cornhole, (h, i) Nintendo Switch sports Chambara and Tennis, (j) balloon volleyball, (k) cup stacking, and (l) FitMi exercises, where FitMi is a commercial sensor system designed to guide upper extremity rehabilitation exercises [17]. One participant was unable to complete several experiments (a, c, f, l) due to an unrelated medical issue. Participants were given a brief introduction to each activity and allowed to practice for a few minutes to ensure their comfort with the task. They were then instructed to perform each activity at a self-paced rate for 10 min. For all activities except ping-pong and cornhole, they were seated. For each activity, we calculated the kurtosis of tilt angle using a sliding window, where we varied the window size (1 min = 3120 samples, 3 min = 9360 samples, and 5 min = 15,600 samples). We shifted the window with a 1 s stride (i.e., 52 data samples) and calculated the mean values of kurtosis across the duration of the activity.

The MiGo contains a six-axis IMU with a sampling frequency of 205 Hz. The device streamed data at 100 Hz to a receiver (nrf52840, Nordic Semiconductor, Trondheim, Norway) connected to a PC. We additionally down-sampled the signals from the study with human subjects from 100 Hz to 52.6 Hz using the scipy.resample_poly function to match the sampling frequency from a previous pilot study of the effectiveness of hand count feedback versus conventional home exercise [18]. In previous research in our lab, we had used the MiGo device to stream accelerometer data at a high rate, and, thus, using the MiGo was convenient to achieve the goals of the human experiments.

To calculate the kurtosis of forearm postural diversity, we first estimated the tilt angle θ, defined as the device’s orientation with respect to gravity, as follows ([13,19]).

(1) $$\theta =\cos^{-1}\left(\frac{a_{z}}{\sqrt{a_{x}^{2}+a_{y}^{2}+a_{z}^{2}}}\right)\;$$

where *a_z_* is defined as the acceleration in the direction perpendicular to the back of the forearm. We then calculated kurtosis offline using rolling windows of durations of 1 min, 3 min, and 5 min using the formula for kurtosis. Note that throughout this paper, we will present “excess kurtosis”, defined as kurtosis minus 3 (i.e., minus the kurtosis of the normal distribution), but refer to it simply as “kurtosis”.

### 2.2. Algorithm for Fast Real-Time Calculation of Kurtosis

Calculating the kurtosis of the tilt angle distribution becomes computationally demanding as the buffer sample size increases. We desire to be able to perform real-time kurtosis computation in our application in order to deliver instantaneous feedback after epochs of higher movement diversity, and this caused us to consider more efficient computation algorithms. Extending the work in [20], we derived a novel kurtosis algorithm (Equation (4)), which we will refer to as “Rolling Sample Kurtosis” (RSK) because it utilizes a rolling sample technique, incorporating values from previous iterations with new sensor data, to enable efficient computation. The real-time computation of kurtosis is performed at every sample.

We first define 𝕍_n_ and 𝕊_n_ as recursive auxiliary variables, as proposed in [20]. 𝕍_n_ (Equation (2)) essentially carries the crucial information of the second central moment, i.e., $\sum_{i=1}^{n}{\left(x_{i}-{\bar{x}}_{n}\right)}^{2}$, and 𝕊_n_ (Equation (3)) carries the crucial information of the third central moment, i.e., $\sum_{i=1}^{n}{\left(x_{i}-{\bar{x}}_{n}\right)}^{3}$.

(2) $$s_{{\left\lceil n\right\rceil }_{k}}^{2}=\frac{V_{{\left\lceil n\right\rceil }_{k}}}{k-1},\;\;n\geq k>1\;V_{{\left\lceil n+1\right\rceil }_{k}}=V_{{\left\lceil n\right\rceil }_{k}}+{(x}_{n+1}-x_{0})\times (x_{n+1}+x_{0}-{\bar{x}}_{{\left\lceil n+1\right\rceil }_{k}}-{\bar{x}}_{{\left\lceil n\right\rceil }_{k}})$$

where

- $s_{{\left\lceil n\right\rceil }_{k}}^{2}$ denotes the sample variance of the most recent *k* observation values, and it can recursively be updated through its corresponding auxiliary variable $V_{{\left\lceil n\right\rceil }_{k}}$ by rolling the observation window to include new values while removing the oldest ones.
  (3) $$S_{{\left\lceil n\right\rceil }_{k}}=\frac{k\times S_{{\left\lceil n\right\rceil }_{k}}}{\left(k-1\right)\left(k-2\right)\times s_{{\left\lceil n\right\rceil }_{k}}^{3}},\;\;n\geq k>2\;S_{{\left\lceil n+1\right\rceil }_{k}}=S_{{\left\lceil n\right\rceil }_{k}}-3\left({\bar{x}}_{{\left\lceil n+1\right\rceil }_{k}}-{\bar{x}}_{{\left\lceil n\right\rceil }_{k}}\right)\times V_{{\left\lceil n\right\rceil }_{k}}+\;{(x}_{n+1}-x_{0})\times \left[\left(x_{0}-{\bar{x}}_{{\left\lceil n\right\rceil }_{k}}\right)\times \left(x_{0}-{2\bar{x}}_{{\left\lceil n+1\right\rceil }_{k}}+{\bar{x}}_{{\left\lceil n\right\rceil }_{k}}\right)+\left(x_{n+1}{-\bar{x}}_{{\left\lceil n+1\right\rceil }_{k}}\right)\times ({x_{n+1}+x_{o}-2\bar{x}}_{{\left\lceil n+1\right\rceil }_{k}})\right]$$

where

- $S_{{\left\lceil n\right\rceil }_{k}}$ represents the sample skewness of the most recent *k* observation values. When the observation window keeps rolling forward, the continuous updating of $S_{{\left\lceil n\right\rceil }_{k}}$will be realized through the recursive calculations of the auxiliary variables $V_{{\left\lceil n\right\rceil }_{k}}$ and $S_{{\left\lceil n\right\rceil }_{k}}$.
  (4) $$K_{{\left\lceil n\right\rceil }_{k}}=\frac{k(k+1)}{\left(k-1)(k-2)(k-3\right)}\times \frac{K_{{\left\lceil n\right\rceil }_{k}}}{s_{{\left\lceil n\right\rceil }_{k}}^{4}},\;\;n\geq k>3\;K_{{\left\lceil n+1\right\rceil }_{k}}=K_{{\left\lceil n\right\rceil }_{k}}-4\left({\bar{x}}_{{\left\lceil n+1\right\rceil }_{k}}-{\bar{x}}_{{\left\lceil n\right\rceil }_{k}}\right)\times S_{{\left\lceil n\right\rceil }_{k}}+6{\left({\bar{x}}_{{\left\lceil n+1\right\rceil }_{k}}-{\bar{x}}_{{\left\lceil n\right\rceil }_{k}}\right)}^{2}\times V_{{\left\lceil n\right\rceil }_{k}}+\;(x_{n+1}-x_{0})\left\{{\left({\bar{x}}_{{\left\lceil n+1\right\rceil }_{k}}-{\bar{x}}_{{\left\lceil n\right\rceil }_{k}}\right)}^{3}+\left[{\left(x_{n+1}-{\bar{x}}_{{\left\lceil n+1\right\rceil }_{k}}\right)}^{2}+{\left(x_{0}-{\bar{x}}_{{\left\lceil n+1\right\rceil }_{k}}\right)}^{2}\right]\times (x_{n+1}+x_{0}-2{\bar{x}}_{{\left\lceil n+1\right\rceil }_{k}})\right\}$$

where

- $K_{{\left\lceil n\right\rceil }_{k}}$ represents the sample kurtosis of the most recent k observation values *x*_n−k+1_, *x*_n-k+2_, …, *x*_n_. When the observation window keeps rolling forward, the continuous update of $K_{{\left\lceil n\right\rceil }_{k}}$ will be based on the recursive calculations of the auxiliary variables $V_{{\left\lceil n\right\rceil }_{k}}$, $S_{{\left\lceil n\right\rceil }_{k}}$, and $K_{{\left\lceil n\right\rceil }_{k}}$.
- *x*_o_ represents the last sensor value from the previous iteration.
- *x*_n+1_ represents the new sensor value, which will update the Rolling Sample Kurtosis.
- ${\bar{x}}_{{\left\lceil n\right\rceil }_{k}}$ stands for the sample mean of the most recent *k* observation values, while ${\bar{x}}_{{\left\lceil n+1\right\rceil }_{k}}$ indicates the updated sample mean after rolling the observation window to include the new value *x*_*n*+1_ while removing the oldest one *x*_*o*_ (i.e., *x*_*n*−*k*+1_).

See Appendix A for the details on the derivation and computational optimization of Equation (4).

We compared the execution speed of the RSK algorithm to the speed of a conventional kurtosis calculation, which iterates through the entire buffer, and to an optimized kurtosis algorithm implemented using Python’s SciPy kurtosis function. The “scipy.stats.kurtosis” function is optimized through the use of vectorized operations provided by NumPy through implementation in C and Fortran in combination with Python and through the incorporation of techniques to ensure numerical stability.

We compared the three methods to calculate kurtosis using Python’s library “timeit”. The Python “timeit” library is a built-in module that provides a simple way to measure the execution time of small code snippets or functions. It is especially useful for the benchmarking and performance testing of Python code since it avoids several common traps for measuring execution times, and it runs the code multiple times to obtain a more accurate estimate of its execution time. Therefore, we ran each one of the three presented kurtosis methods 100 times to obtain a better estimate of execution time.

For all three methods, the buffer was a list of tilt angles, initialized with *None* elements, which are built-in in Python and are faster than using numpy.nan. Moreover, using a list instead of an array in Python can be slightly more efficient in some cases.

### 2.3. Experiment 2: Robotic Validation of the Rolling Sample Kurtosis Calculation as Implemented on a Smartwatch

In the human experiments described above, we used the MiGo device to acquire accelerometer data. However, MiGo is a research device developed by Flint Rehabilitation Devices (Flint Rehabilitation Devices, Irvine, CA, USA) [17], and we did not have the capability of modifying its firmware to implement real-time kurtosis. So, for implementing and testing real-time kurtosis, we chose the Samsung watch, which has several desirable features, including a low-power CPU capable of performing real-time data processing, sophisticated visual display and vibrotactile feedback capabilities, and excellent accessibility and usability. To validate the RSK algorithm, we implemented it on a commercial smartwatch. We chose the Samsung Galaxy Watch 4 due to its low cost and widespread accessibility. We developed a Java-based (Version 8 Update 311) kurtosis application for this Wear OS (API Level 33, Android 13) device using Android Studio.

The sensor event delivery rate, or sampling rate, dictates the frequency at which sensor data are transmitted to the application through the “onSensorChanged()” callback process. In this work, we chose a sampling frequency of ~50 Hz to align with previous work [13] and because it is sufficient to capture human movement [21].

**Figure 2:**
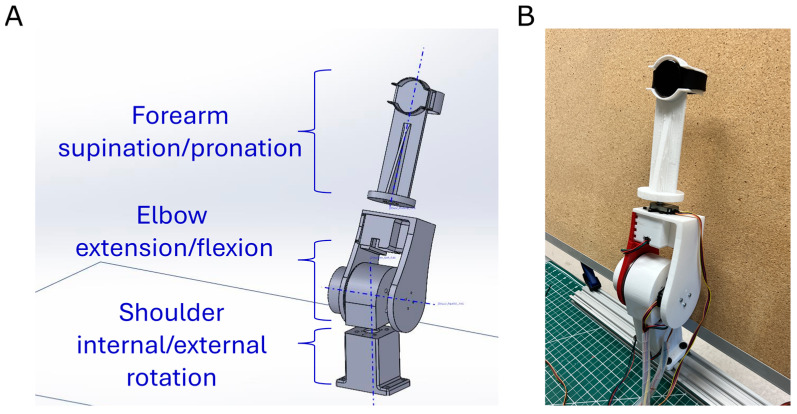
(**A**): A 3D model designed using SolidWorks. (**B**): The spherical wrist, 3D-printed and assembled with the smartwatch “worn” at the end-effector.

For the validation experiments, we strapped the Samsung watch to the wrist of a custom-built, three-degree-of-freedom (DOF) robot arm that allowed us to create human-like movements with pre-specified levels of movement diversity. The robot was intended to simulate human forearm biomechanics when the elbow is resting on a table or an armrest, or, equivalently, when the translation of the elbow is small during arm use. The three revolute joints imitate the effect of shoulder internal/external rotation (“rot”), elbow extension/flexion (“ext”), and forearm supination/pronation (“sup”) (Figure 2).

We printed the robot parts using a 3D printer (Bambu Lab X1-Carbon Combo 3D Printer, Bambulab USA Inc., Austin, TX, USA). We used two high-torque stepper motors (STEPPERONLINE 19:1 NEMA17, STEPPERONLINE Inc., New York, NY, USA) with drivers (TB6600 Stepper Motor Drivers, SZCY LLC, Maoming, China) equipped with a 19:1 gearbox to control the “rot” and “ext” with a step resolution of 0.094°. For slow speeds, the “rot” and “ext” steppers were configured to a 1/16th step size, achieving a maximum speed of 15°/s and an acceleration of 15°/s^2^. For fast speeds, these steppers were set to a 1/4th step size, reaching a peak speed of 90°/s with an acceleration of 90°/s^2^.

A Nema17 stepper motor (STEPPERONLINE Nema 17, STEPPERONLINE Inc., New York, NY, USA), also with a TB6600 driver, was used for the “sup” DOF, with a step resolution of 1.8° and drivers set at a 1/32nd step size both for the slow and fast speeds. At slow speeds, the motor achieved 30°/s with an acceleration of 30°/s^2^ while at fast speeds, it reached up to 180°/s with 180°/s^2^.

We used relative encoders (CUI Devices AMT102-V, AutomationDirect.com, Atlanta, GA, USA) to detect the angular position of each joint and a microcontroller (Arduino Mega, Arduino S.R.L, Torino, Italy) as the controller and data acquisition system.

We used the Unified Robotics Description Format (URDF) [22] to model the robot and to represent its kinematic structure and visual appearance. We used the Robotics Toolbox (RTB10.4) from Peter Corke [23] to describe the robot as a serial-link arm-type robot using the Denavit–Hartenberg parameters (Table 1). The joint variables were numbered progressively starting from 4 since the robot resembles a spherical wrist, which is typically thought of as mounted on a three-DOF arm of a six-DOF manipulator [24].

The complete forward kinematics model T for this robot is:

(5) $$T=\left[\begin{array}{cccc} c4\times c5\times c6-s4\times s6 & -c6\times s4-c4\times c5\times s6 & c4*s5 & d6\times c4\times s5\\ c4\times s6+c5\times c6\times s4 & c4\times c6-c5\times s4\times s6 & s4*s5 & d6\times s4\times s5\\ -c6\times s5 & s5\times s6 & c5 & d4+d6\times c5\\ 0 & 0 & 0 & 1 \end{array}\right]$$

The upper left 3 × 3 array of (5), i.e., T(1:3, 1:3), represents the rotation matrix R with respect to the origin frame, and the first three elements of the last column, i.e., T(1:3, 4), give us the position of the end-effector in x-y-z. We calculated the ground truth sensor tilt angles from the end-effector’s pose, applying the rotation matrix R to ${\vec{a}}_{home}=\left[1;0;0\right]$ to estimate the watch’s tilt angle θ for every possible configuration of joint positions.

We used the robot to implement six movement trajectories with known values of forearm postural kurtosis (see Appendix B). We chose four of these trajectories to roughly mirror human forearm motions during simple activities and thus named them: shuffling cards, cup stacking, arm wrestling, and handshaking. The fifth trajectory, named exploration, was devised to encompass the full range of joint positions by driving each motor to its maximum extent. The final trajectory, referred to as simulated normal, was crafted to exhibit a distribution of tilt angles with a kurtosis value closely matching a mesokurtic distribution.

The microcontroller dispatched the predefined trajectories to the stepper motors via the Arduino AccelStepper library (1.64.0). Each trajectory was simulated fifteen times, and the experimental kurtosis was calculated as the average of these runs. The Samsung watch recorded the resulting tilt angle values within its internal storage, utilizing an SQL database. At the same time, a secondary board (Arduino MEGA) saved the encoder values from each robot’s joint to a .txt file using CoolTerm (2.2.0), a Software for communicating with serial devices via USB serial communication. The Arduino Mega does not support parallel programming since its microcontroller is single-core and can only execute one instruction at a time; however, we needed to run three tasks in parallel, one for each encoder. Thus, we used a class of RTOS (Real-Time Operating System) called FreeRTOS, which can be run on 8/16-bit microcontrollers such as Arduino. RTOS is designed to run applications with very precise timing and a high degree of reliability, and it also helps in multi-tasking with a single core, allowing us to run the three encoder functions/tasks concurrently. Additionally, we used Semaphores and Mutex for synchronization, resource management, and protecting resources from corruption.

As the robot moved, it “wore” the Samsung smartwatch on its wrist. We filtered the measured acceleration with a Fast Fourier Transform (FFT)-based, low-pass filter with a cutoff frequency of 1 Hz and used the tilt angle estimation approach shown in Equation (1) as input into the kurtosis algorithm.

To align the watch and robot data, the following procedure was used. At the beginning of each movement, the robot was positioned in its home position, as shown in Figure 2B. When in the home position, the watch began saving data. Subsequently, the robot started its movement. We aligned the watch’s data to the robot data by identifying the start of the movement in the watch data.

## 3. Results

### 3.1. Different Therapeutic Activities Generated a Wide Range of Kurtosis Values

**Figure 3:**
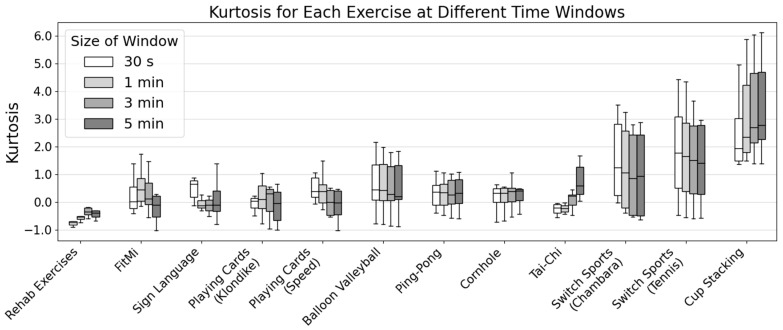
Kurtosis of tilt angle for 12 different tasks at 0.5, 1, 3, and 5 min time windows across participants. The box color represents the window size: white (30 s), light gray (1 min), gray (3 min), and dark gray (5 min). The solid black line within each box represents the median value, while the whiskers extend to the minimum and maximum values within 1.5 times the interquartile range.

A group of seven unimpaired individuals performed 12 activities, similar to what might be used for upper extremity rehabilitation or during recreational activities after neurologic injury (Figure 3). We found that the activities produced median kurtosis values that ranged between −0.39 and 2.77 for the 5 min analysis window (Figure 3). Using shorter windows of 1 and 3 min had only a small effect on the measured kurtosis (Figure 3). This indicates that different activities produced more tailed (k < 0) or more peaked (k > 0) distributions than the normal distribution (k = 0).

The activity that yielded the lowest value for kurtosis was the book of rehabilitation exercises. That is, this activity had the least peaked forearm posture distribution and, thus, the greatest diversity of forearm posture, as defined using kurtosis. The activity that yielded the highest value for kurtosis was cup stacking since the distribution of tilt angles was highly peaked for this activity, consistent with a narrow angular range of forearm movement. In general, there was relatively small intersubject variability in kurtosis for each activity, except for Switch sports (Tennis and Chambara) and cup stacking, which displayed a larger interquartile range, indicating higher, cross-subject variability in the data.

### 3.2. The Rolling Sample Kurtosis (RSK) Algorithm Accelerated Computation Speed

**Figure 4:**
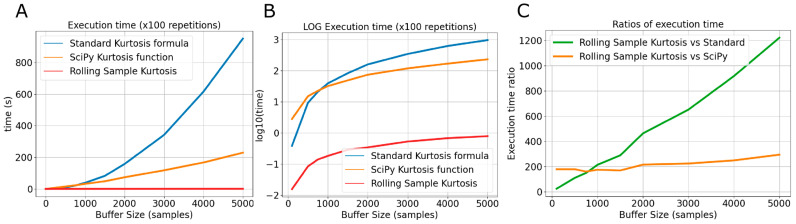
(**A**): Increasing the window size n increases the execution time. We fitted a 2nd-order polynomial to the data, resulting in an equation for standard kurtosis of (3.7280 × 10^−5^x^2^ + 0.0016x + 0.8461), for the SciPy function (3.3760 × 10^−6^x^2^ + 0.0288x − 0.3913), and for the Rolling Sample Kurtosis (−8.0800 × 10^−9^x^2^ + 0.0002x − 0.0062). (**B**): Logarithmic plot of the same execution times. (**C**): The ratio of execution speed vs. buffer size when comparing the Rolling Sample Kurtosis approach against the standard method (green) and the SciPy function (in orange).

We executed the kurtosis computation 100 times in Python using the “timeit” function on varying sample buffers of size *n* for three calculation methods: standard kurtosis formula, SciPy kurtosis function, and RSK (Figure 4). RSK accelerated the computation. For example, when applied to a sample window of *n* = 5000, the average execution time for the standard kurtosis formula was 9.5 s, compared to 2.28 s for the SciPy kurtosis function and 7.77 ms for the Rolling Sample Kurtosis method. Across buffer sizes, the saving compared to the optimized SciPy kurtosis function was ~200-fold for sample sizes < 3000. Due to the recursive nature of the algorithm, the RSK computational time increased more slowly with buffer size as well, reaching a ~300-fold gain against the SciPy approach at a buffer size of 5000 (Figure 4).

### 3.3. Robotic Validation of the Rolling Sample Kurtosis Calculation as Implemented on a Smartwatch

We implemented six movement sequences with varying expected values of kurtosis using the custom-built robot. The robot “wore” a smartwatch on its wrist during these movements, and the smartwatch calculated kurtosis using the RSK algorithm.

**Figure 5:**
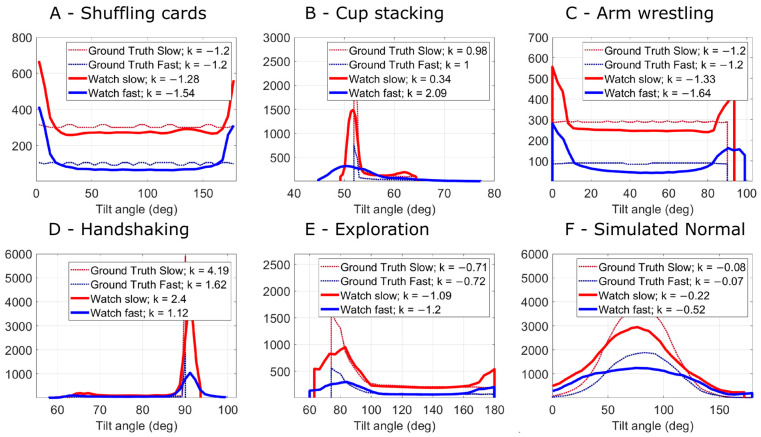
Histograms of smartwatch tilt angles representing the six tested trajectories with different values of kurtosis. Shown are the ground truth (calculated directly from the trajectories) and the estimated tilt angle (from the smartwatch) at two motor speeds. Refer to Appendix C for an explanation of the peaks at 0° and 180° in plots A and C. Please note that the ground truth values for slow and fast speeds are nearly identical in each pair, with the exception of plot D—handshaking. In this particular plot, the trajectory at fast speeds deviates slightly from that at slow speeds; thus, the ground truths are different. This was due to a speed limitation in the supination motor for the fast speeds.

**Figure 6:**
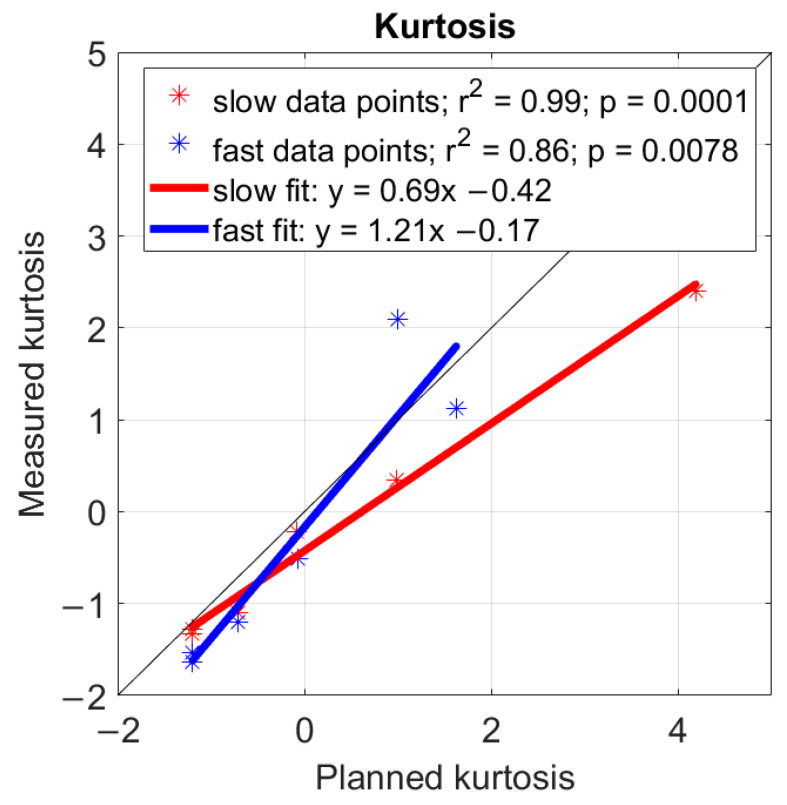
Planned and measured kurtosis of the different trajectories. A normal distribution has a kurtosis of K = 0 and values lower than 0 indicate a more spread-out distribution. The thin, black line has a slope of 1. The planned kurtosis value for the “handshaking” fast movement (1.62) was lower than for the slow movement (4.19) due to a speed limitation in the supination motor for the fast movement.

First, we observed that the distribution of tilt angles measured with the smartwatch was comparable with the distribution of tilt angles calculated from the robot trajectories (Figure 5), both for slow and faster speeds, although there were some errors. Second, we observed that kurtosis calculated from the smartwatch-estimated tilt angles using the RSK algorithm was highly correlated with planned kurtosis for the trajectories for slow motor speeds (r^2^ = 0.99, *p* = 0.0001; Figure 6) and for faster speeds (r^2^ = 0.86, *p* = 0.0078; Figure 6), although the slope was under 1 for the faster speeds, indicating that we underestimated kurtosis as faster speeds.

**Figure 7:**
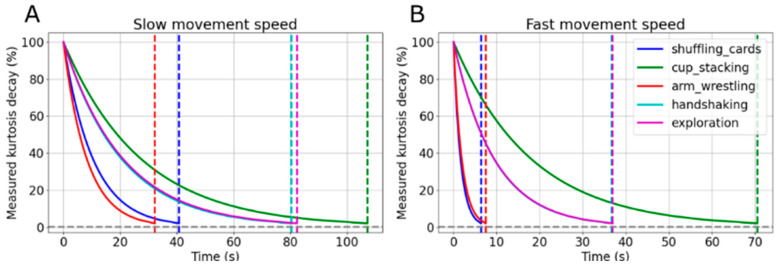
The measured kurtosis decay for each experiment as a function of time. Shown are exponential fits to the envelopes of the real-time, measured kurtosis. The raw data for these experiments are provided in Appendix D. (**A**): Slow speed; (**B**): faster speed. The vertical dashed lines show the times when each activity reaches a steady state, considered to be within 2% of the actual kurtosis value (horizontal dashed line).

Finally, an interesting observation from the experiment with human participants described above is that the kurtosis estimate remained approximately constant for windows of 1, 3, and 5 min. To determine what window size is adequate for accurately estimating the kurtosis of epochs of human-like movement trajectories during repeated activities, we repeated the experiment with the data from the robot. Due to the cyclic nature of the trajectories, we expected that the kurtosis values would stabilize after a certain period. We plotted the kurtosis as a function of the window size, determined the envelope of the resulting function, and fitted an exponential decay model to this envelope. Using the decay parameter derived from this model, we estimated the time required for the kurtosis to diminish to a specified percentage of its initial value. As illustrated in Figure 7, the kurtosis computed with RSK converged to 2% of the planned kurtosis value of each experiment within less than 100 s for lower speeds and under 70 s for higher speeds. When an activity is repeated, then the kurtosis converges in a few minutes.

## 4. Discussion

These results represent needed steps toward the goal of providing wearable feedback about QOME (i.e., the quality of the movement experience). First, in an experimental study with young, unimpaired adults, we verified that different movement tasks produce a range of forearm tilt angle kurtosis values. Thus, asking people to seek out and perform different movement tasks can theoretically serve as a means to increase daily UE movement diversity, as measured with kurtosis. Second, we derived a recursive algorithm (Rolling Sample Kurtosis—RSK) for the real-time calculation of kurtosis on a smartwatch and verified that it dramatically improves computation speed. Third, we embedded the RSK algorithm on a commercial smartwatch and validated its accuracy using a robotic simulator that “wore” the smartwatch. We now discuss these results and then study limitations and directions for future research.

### 4.1. Daily Activities and Forearm Postural Kurtosis

In a previous study with persons with UE impairment after stroke, we found that individuals with severe movement impairment displayed lower levels of forearm postural diversity, as quantified with kurtosis [13]. This observation has motivated us to work toward the goal of providing a wearable sensing tool for individuals post-stroke that they can use to encourage forearm postural diversity in home rehabilitation. The present study indicates that engaging in different activities can potentially improve diversity, as detected with kurtosis—the range of activities we tested had a wide range of forearm postural kurtosis. Further, it was interesting to us that engaging in a typical home rehabilitation program produced the most UE movement diversity as quantified by kurtosis. This provides a unique validation of the conventional approach to home rehabilitation in terms of verifying its value for promoting movement diversity, at least when those tasks are performed by unimpaired individuals. Future research should confirm that different activities modulate kurtosis in the case where those activities are performed by people with movement impairment after a stroke.

Notably, kurtosis values remained relatively stable across the 1, 3, and 5 min windows for the tasks we studied, indicating that these metrics can be effectively captured even within short periods for repetitive daily activities. Our follow-up experiment with the robotic arm that simulated different movement trajectories also confirmed that kurtosis estimates can converge in approximately ~1 min for repetitive movement tasks. Incorporating additional data points into the distribution enhances the smoothness of the kurtosis estimate by improving the stability of the statistical estimates. This is achieved by averaging out anomalies and yielding a distribution that more accurately reflects the true population. Nevertheless, reasonable accuracy is possible with 1 min windows. We found that kurtosis converges in a few minutes when a movement activity is repeated.

Knowing this convergence rate is helpful, as many daily work, leisure, and exercise activities are repetitive in nature. Our future goal is to provide feedback to participants wearing the watch when an activity they are engaging in is more diverse. For example, one could potentially have a smartwatch vibrate after it observes a movement epoch of approximately 1 min with unusually low kurtosis. These vibrations could serve to notify the user that they are “doing something desirable” in terms of exploring new movements. To achieve this, we need to specify a sample window size that allows us to calculate kurtosis and deliver feedback as promptly as possible. But we do not want to deliver feedback so fast that it is confusing to the individual concerning what triggered the feedback. Knowing the convergence time is approximately 1 min for repetitive activities gives an idea of an appropriate window size for many of our daily work, leisure, and exercise activities. We note, however, that if activities are not being repeated, it is unlikely kurtosis will converge as demonstrated here.

### 4.2. Recursive Algorithm Accelerates Computation Speed

Providing real-time, wearable feedback about kurtosis requires being able to implement the computation on a smartwatch. We derived a novel, recursive algorithm for calculating kurtosis, the Rolling Sample Kurtosis method. RSK sped up kurtosis computation by a factor of ~200 compared to the SciPy function on an average basis for window sizes < 3000. Since the RSK algorithm operates more quickly, it potentially allows for a higher sampling frequency to be utilized, which could improve estimation accuracy. As a side note, other sensing applications besides QOME-based movement rehabilitation may find the RSK algorithm useful for computing these higher-order moments of statistical distributions, which is a need, for example, in machine learning for IoT devices [20].

### 4.3. Robotic Validation of Smartwatch Algorithms

A key problem in implementing wearable sensing algorithms is the difficulty in assessing ground truth. Implementing a kurtosis algorithm on a smartwatch is feasible; however, ensuring its accuracy is challenging. We previously used a simple robotic device to evaluate an algorithm for detecting finger movement using a wearable, magnetic sensing system [18]. Here, we followed a similar methodology, using a custom-built robotic device that mimics the human forearm to create movement patterns with known kurtosis values. This allowed us to verify the accuracy of the RSK algorithm as implemented on a smartwatch.

The kurtosis estimate was less accurate at faster robot speeds, generally tending to underestimate the actual kurtosis. We speculate this is due to a greater error in estimating tilt angle at higher speeds. Thus, an important direction for future research is the issue of how tilt angle estimation affects kurtosis estimates. The method we used to estimate forearm tilt angle is computationally simple and widely used [19] but it assumes the accelerometer is not moving, as it is based solely on estimating the gravity vector orientation in static conditions. When the accelerometer moves, it will introduce errors into the estimate, with errors increasing at higher speeds. We tested two movement speeds with the robot (a baseline slow speed at ~0.06 Hz and the faster movements at ~0.18 Hz), finding excellent kurtosis estimation accuracy for both. However, these speeds are in the lower range of human UE movement speeds. According to the study in [25], the frequency spectral analysis of wrist movements during activities of daily living revealed that the predominant frequency components ranged from 0.48 to 2.47 Hz, with a mean value of 1 Hz, a finding consistent with the observation that power in a UE tracking task was mostly found up to 1 Hz [26]. Additionally, research by the authors of [27] on UE reaching after stroke found predominant power in the movement signal up to ~0.5 Hz. Future work will use a robot capable of higher movement speeds to understand the effect of speed on tilt angle and kurtosis estimation. Other tilt angle estimation approaches are possible [28].

### 4.4. Toward Clinical Application of QOME-Based Feedback

Exploration and challenge are fundamental principles of both motor learning and rehabilitation, yet existing wearable sensors simply count the quantity of movement. Future work should test the hypothesis that quantitative assessments of the quality of the movement experience—as captured, for example, by forearm posture diversity—are beneficial in designing wearable feedback systems for post-stroke rehabilitation because they can encourage patients to practice specific exercises or activities that challenge the patient in a desirable way. For example, with a real-time measure of QOME implemented on a smartwatch, it would be possible to test if the smartwatch-based feedback of QOME could be used to encourage people to explore new movement patterns, thereby challenging their motor system and promoting movement recovery. The results presented in this paper move us a step closer to implementing a clinical trial that tests this possibility.

### 4.5. Other Potential Uses of Kurtosis

By definition, in statistics, kurtosis is a powerful tool together with skewness to measure the normality of datasets [29]. Naturally, kurtosis has been employed for application-agnostic research on investigating the characteristics of real-world data samples [30]. In practice, the kurtosis-based data characterization has shown its unique value in a wide range of application domains. For example, kurtosis is proposed as a better indicator to overcome the problem of galaxy biasing in large-scale cosmic fields [31]; systematic kurtosis is incorporated into an asset pricing model to estimate the investment risks and returns [32]; mean kurtosis offers an improved sensitivity in detecting developmental and pathological changes in neural tissues as compared to conventional diffusion tensor imaging [33]; diffusional kurtosis is investigated as a potential metric for clinical stroke evaluation [34]; and in fact, diffusion kurtosis imaging (DKI) has been developed as a novel technique for evaluating pathology throughout the body in various clinical settings [35]. In these various applications, the real-time implementation of kurtosis derived here could possibly find use.

### 4.6. Limitations

Limitations of the current study include the following. First, as mentioned in the Introduction, the kurtosis of forearm tilt can distinguish persons with severe and moderate impairment after stroke but not persons with moderate and mild impairment. Thus, other measures of the QOME for less impaired individuals besides kurtosis are likely needed. Second, we studied unimpaired participants in the experimental study of how different activities affect kurtosis. Impaired persons may have greater difficulty varying forearm tilt diversity during different activities. Future work should study this issue in people with a range of impairment levels. Third, our estimates for convergence time for estimating kurtosis are for repetitive activities, but not all movement activities are repetitive. Fourth, the robot we developed could only move at slow speeds. Investigating the effect of higher movement speed on kurtosis calculation is an important future direction and will likely depend on implementing more sophisticated forearm posture estimation algorithms. Finally, the ultimate validation of QOME feedback will require a randomized controlled clinical trial.

## 5. Conclusions

This study found that different movement tasks have different kurtosis values; derived a novel, recursive algorithm that dramatically improves the computation speed of kurtosis; and used a robot arm to demonstrate that the algorithm accurately computes kurtosis in real time. We are moving forward now to using the real-time kurtosis algorithm on a commercial smartwatch to provide QOME feedback for stroke rehabilitation.

## Figures and Tables

**Table 1:** The robot defined with the Denavit–Hartenberg parameters.

Link i	ai	αi	di	qi
4	0	−pi/2	0.056	q4
5	0	pi/2	0.000	q5
6	0	0	0.300	q6

**Figure A1:**
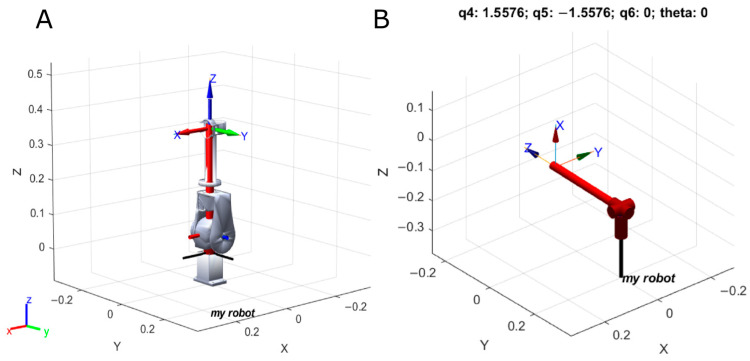
(**A**): Simulation of the spherical wrist in MATLAB using both the URDF and DH parameters. Note that we are interested in the orientation of the *x*-axis from the end-effector where the watch will be placed. (**B**): The robot rotated with a tilt angle theta of 0 rad (the *x*-axis is oriented parallel to the world’s normal).

**Figure A2:**
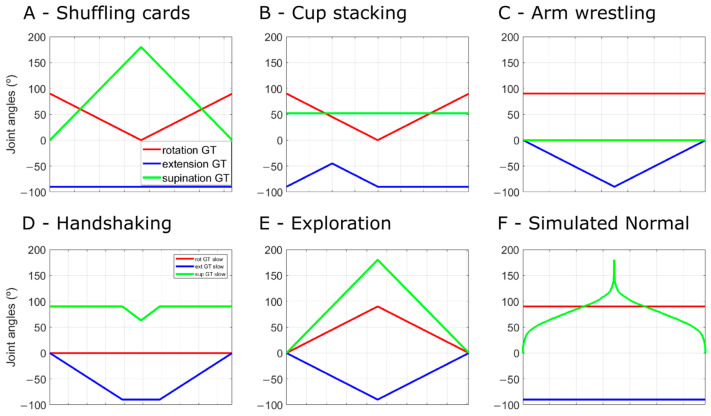
The trajectories from each of the 3-stepper motors; rotation (red), extension (blue), supination (green).

**Figure A3:**
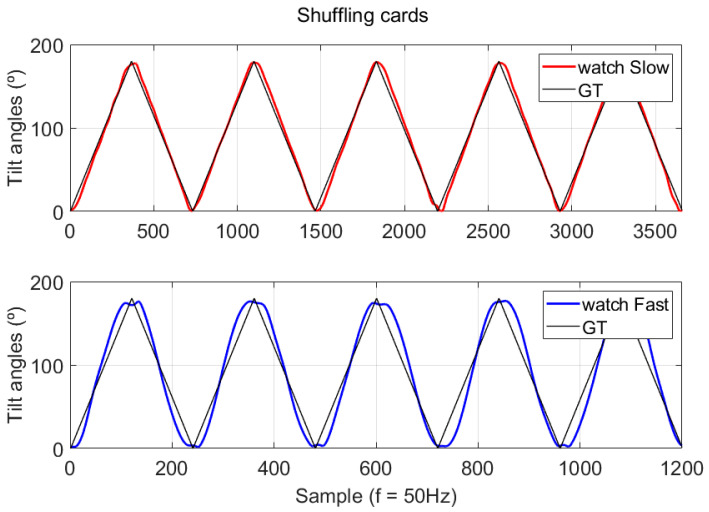
The tilt angles from the first 5 repetitions of the shuffling cards activity. The slow speed is indicated in red, and the faster speed is represented in blue. “GT” denotes ground truth.

**Figure A4:**
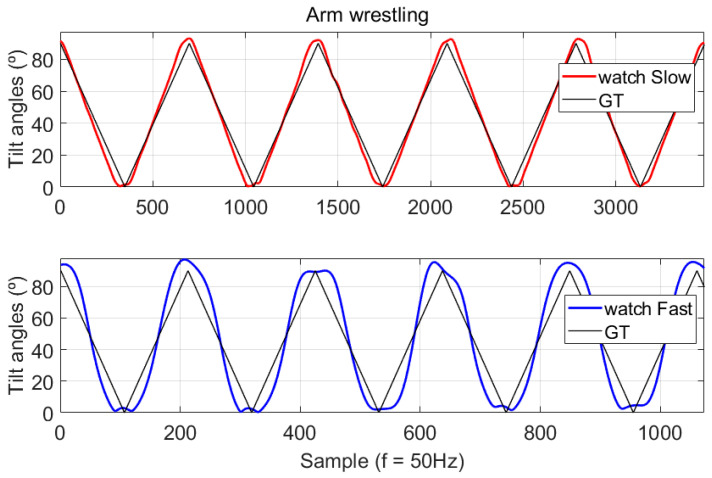
The tilt angles from the first 5 repetitions of the arm wrestling activity. The slow speed is indicated in red, and the faster speed is represented in blue. “GT” denotes ground truth.

**Figure A5:**
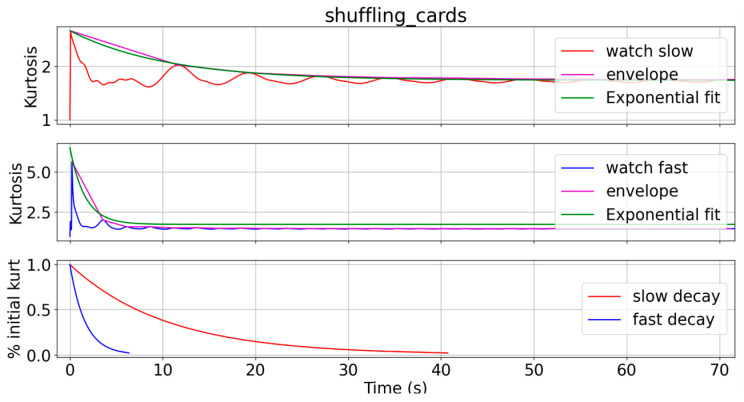
The “shuffling cards” trajectory.

**Figure A6:**
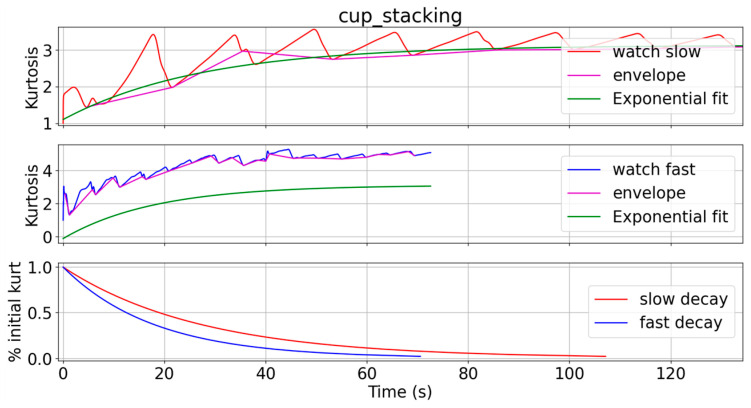
The “cup stacking” trajectory.

**Figure A7:**
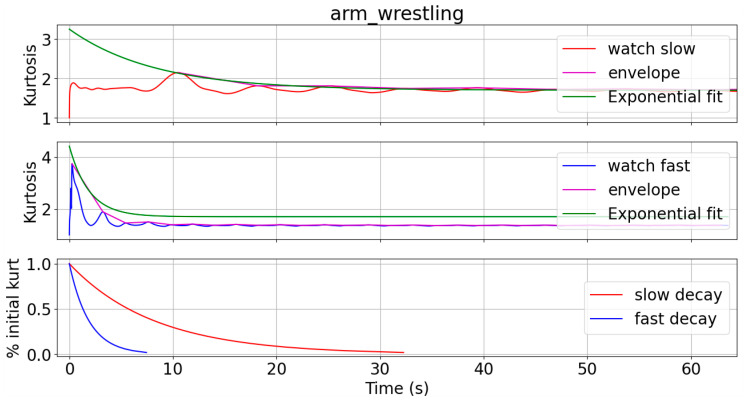
The “arm wrestling” trajectory.

**Figure A8:**
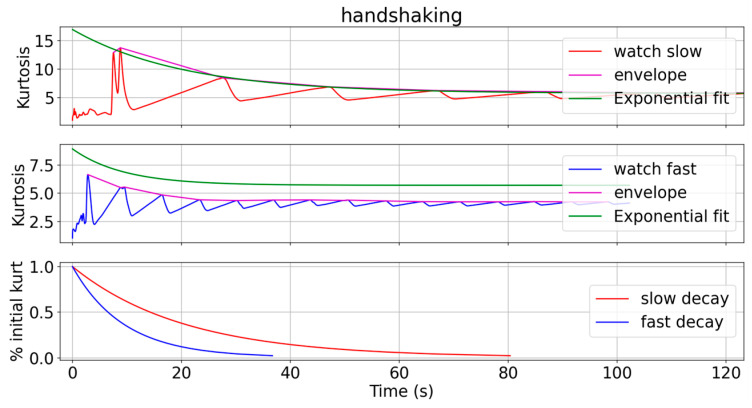
The “handshaking” trajectory.

**Figure A9:**
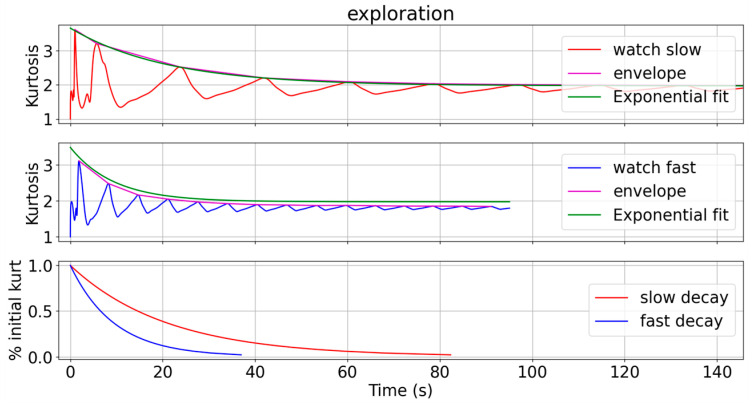
The “exploration” trajectory.

## Data Availability

The original data presented in the study are openly available on GitHub at https://github.com/gcornella/RealTime-Kurtosis (accessed on 21 June 2024).

## Appendix A

The Rolling Sample Kurtosis (Equation (4)) implements the following concepts to improve performance:

- The maximization of the usage of intermediate results to avoid repeating the same calculations.
- The minimization of the multiplication operations to reduce the calculation overhead, e.g., (*x*_n+1_ + *x*_0_ -2${\bar{x}}_{{\left\lceil n+1\right\rceil }_{k}}$) in (2) should be calculated in the way of [(*x*_n+1_ − ${\bar{x}}_{{\left\lceil n+1\right\rceil }_{k}}$) + (*x*_0_ − ${\bar{x}}_{{\left\lceil n+1\right\rceil }_{k}}$)], which also maximizes the usage of intermediate results if (*x*_n+1_ − ${\bar{x}}_{{\left\lceil n+1\right\rceil }_{k}}$) and (*x*_0_ − ${\bar{x}}_{{\left\lceil n+1\right\rceil }_{k}}$) are already calculated beforehand.
- The avoidance of exponential calculations. For example, we can use a temporary variable to save the intermediate result, e.g., dif = (${\bar{x}}_{{\left\lceil n+1\right\rceil }_{k}}$ − ${\bar{x}}_{{\left\lceil n\right\rceil }_{k}}$). As such, (${\bar{x}}_{{\left\lceil n+1\right\rceil }_{k}}$ − ${\bar{x}}_{{\left\lceil n\right\rceil }_{k}}$)^2^ can be calculated as dif2 = dif*dif, and (${\bar{x}}_{{\left\lceil n+1\right\rceil }_{k}}$ − ${\bar{x}}_{{\left\lceil n\right\rceil }_{k}}$)^3^ can be calculated as dif3 = dif*dif2.

**Algorithm A1**. Rolling Sample Kurtosis	
**Input**: The most recent tilt angle calculated at every sensor event using the accelerometer values (newValue—Equation (1)), the previously deleted tilt angle (poppedValue), the buffer index counter (i), and the previous results from the last iteration (oldMean, oldM2, oldM3, oldM4, and oldKurtosis)	
**Output**: The newly updated results (newMean, newM2, newM3, newM4, and newKurtosis)	

newValue	→ a new tilt angle is generated
**if** (i == 1 **and** poppedValue == −1) **then**	→ when receiving the first calculated tilt angle
newMean = newValue	→ update the mean
newM2 = newM3 = newM4 = newKurtosis = 0	→ initialize variables
**else if** (poppedValue == −1) **then**	→ Incremental approach (the buffer is not full yet)
delta = newValue - oldMean	
delta_n = delta/i	
delta_n2 = delta_n*delta_n	
term1 = delta * delta_n * (i − 1)	
newMean = oldMean + delta_n	→ Cumulative Sample Mean (CSM)
dif1 = newValue – newMean	→ Intermediate assignations
dif2 = newMean − oldMean	
newM2 = oldM2 + dif1*(_newValue - oldMean)	→ Cumulative Sample Variance (CSV)
newM3 = oldM3 − 3*dif2*oldM2 + (newM2 − oldM2)*(dif1 − dif2)	→ Cumulative Sample Skewness (CSS)
newM4 = oldM4 + term1 * delta_n2 *(i*i − 3*i + 3) + 6*delta_n2*oldM2 - 4*delta_n*oldM3	→ Cumulative Sample Kurtosis (CSK)
newKurtosis = i * newM4 / (newM2 *newM2)	→ Kurtosis
**else**	→ Rolling approach (the buffer is full)
dif3 = newValue - poppedValue	→ Intermediate assignations
newMean = oldMean + dif3/MAX_SIZE	→ Rolling Sample Mean (RSM)
dif4 = poppedValue − newMean	→ Intermediate assignations
dif5 = newValue − newMean	
dif6 = newMean − oldMean	
dif7 = _poppedValue − oldMean	
sum1 = dif4 + dif5	
newM2 = oldM2 + dif3*(dif5 + dif7)	→ Rolling Sample Variance (RSV)
newM3 = oldM3 − 3*dif6*oldM2 + dif3*(dif7*(dif4 − dif6) + dif5*sum1)	→ Rolling Sample Skewness (RSS)
dif6_2 = dif6*dif6	→ to avoid exponential calculations
newM4 = oldM4 − 4*dif6*oldM3 + 6*dif6_2*oldM2 + dif3*(dif6*dif6_2 + sum1*(dif5*dif5 + dif4*dif4))	→ Rolling Sample Kurtosis
newKurtosis = MAX_SIZE * newM4 / (newM2 * newM2)	→ Kurtosis
**end if**	
_mean = newMean _M2 = newM2 _M3 = newM3 _M4 = newM4 _kurtosis = newKurtosis	→ update the old values with the new ones
**return** newMean, newM2, newM3, newM4, **newKurtosis**	

## Appendix B

For all trajectories, the robot’s “rot” joint was allowed to vary between 0° and 90° to align with the human right forearm’s motion range. Similarly, the “ext” spanned from 0 to −90°, and the “sup” was modulated from 0° to 180°, to closely replicate the entirety of possible movements.

## Appendix C

Notice that the experimental tilt angles calculated by the watch display a higher frequency of values around the minimum (0°) and maximum (180°) ranges (Figure A3). This occurs primarily because the robot, which is built with stepper motors, cannot change direction immediately, and the watch records more points at the extremes. Additionally, the applied filtering smooths the signal at its peaks, leading to a histogram plot with more values at these extremes, as illustrated by the peaks in Figure 5A.

The same phenomenon can be seen with the arm wrestling activity (Figure A4).

## Appendix D

The following figures illustrate the convergence of kurtosis over time. As the time window extends, the kurtosis values tend to stabilize. The upper subplots (slow speed) and mid subplots (fast speed) present exponential fits (green lines) to the envelopes (magenta lines) of the real-time measured kurtosis. The bottom subplots display the decay of measured kurtosis for each experiment over time, with the slow speed shown in red and the fast speed shown in blue.

## References

[1] Bravata D.M., Smith-Spangler C., Sundaram V., Gienger A.L., Lin N., Lewis R., Stave C.D., Olkin I., Sirard J.R. (2007). Using pedometers to increase physical activity and improve health: A systematic review. JAMA.

[2] Adans-Dester C.P., Lang C.E., Reinkensmeyer D.J., Bonato P. (2022). Wearable Sensors for Stroke Rehabilitation. Neurorehabilitation Technology.

[3] Okita S., Reinkensmeyer D. (2023). Forearm Postural Diversity and Complexity: Targets for Wearable Feedback after Stroke?.

[4] Balasubramanian S., Melendez-Calderon A., Burdet E. (2012). A Robust and Sensitive Metric for Quantifying Movement Smoothness. IEEE Trans. Biomed. Eng..

[5] DeJong S.L., Schaefer S.Y., Lang C.E. (2012). Need for speed: Better movement quality during faster task performance after stroke. Neurorehabil. Neural Repair.

[6] Cirstea M.C., Levin M.F. (2000). Compensatory strategies for reaching in stroke. Brain J. Neurol..

[7] Ellis M.D., Lan Y., Yao J., Dewald J.P.A. (2016). Robotic quantification of upper extremity loss of independent joint control or flexion synergy in individuals with hemiparetic stroke: A review of paradigms addressing the effects of shoulder abduction loading. J. Neuroeng. Rehabil..

[8] Krakauer J.W., Kitago T., Goldsmith J., Ahmad O., Roy P., Stein J., Bishop L., Casey K., Valladares B., Harran M.D. (2021). Comparing a Novel Neuroanimation Experience to Conventional Therapy for High-Dose Intensive Upper-Limb Training in Subacute Stroke: The SMARTS2 Randomized Trial. Neurorehabil. Neural Repair.

[9] Huang F.C., Patton J.L. (2013). Augmented Dynamics and Motor Exploration as Training for Stroke. IEEE Trans. Biomed. Eng..

[10] Huang F.C., Patton J.L. Individual patterns of motor deficits evident in movement distribution analysis. Proceedings of the 2013 IEEE 13th International Conference on Rehabilitation Robotics (ICORR).

[11] Rast F.M., Labruyère R. (2020). Systematic review on the application of wearable inertial sensors to quantify everyday life motor activity in people with mobility impairments. J. NeuroEngineering Rehabil..

[12] Cinnera A.M., Picerno P., Bisirri A., Koch G., Morone G., Vannozzi G. (2024). Upper limb assessment with inertial measurement units according to the international classification of functioning in stroke: A systematic review and correlation meta-analysis. Top. Stroke Rehabil..

[13] Okita S., de Lucena D.S., Reinkensmeyer D. (2023). Movement Diversity and Complexity Increase as Arm Impairment Decreases after Stroke: Quality of Movement Experience as a Possible Target for Wearable Feedback.

[14] See J., Dodakian L., Chou C., Chan V., McKenzie A., Reinkensmeyer D.J., Cramer S.C. (2013). A Standardized Approach to the Fugl-Meyer Assessment and Its Implications for Clinical Trials. Neurorehabil. Neural Repair.

[15] Mishra P., Pandey C.M., Singh U., Gupta A., Sahu C., Keshri A. (2019). Descriptive statistics and normality tests for statistical data. Ann. Card. Anaesth..

[16] Zondervan D.K., Friedman N., Chang E., Zhao X., Augsburger R., Reinkensmeyer D.J., Cramer S.C. (2016). Home-based hand rehabilitation after chronic stroke: Randomized, controlled single-blind trial comparing the MusicGlove with a conventional exercise program. J. Rehabil. Res. Dev..

[17] Swanson V.A., Johnson C., Zondervan D.K., Bayus N., McCoy P., Ng Y.F.J., Schindele B.J., Reinkensmeyer D.J., Shaw S. (2023). Optimized Home Rehabilitation Technology Reduces Upper Extremity Impairment Compared to a Conventional Home Exercise Program: A Randomized, Controlled, Single-Blind Trial in Subacute Stroke. Neurorehabil. Neural Repair.

[18] de Lucena D.S., Rowe J., Chan V., Reinkensmeyer D.J. (2021). Magnetically Counting Hand Movements: Validation of a Calibration-Free Algorithm and Application to Testing the Threshold Hypothesis of Real-World Hand Use after Stroke. Sensors.

[19] Pedley M. (2013). Tilt Sensing Using a Three-Axis Accelerometer. Free. Semicond. Appl. Note.

[20] Li Z., Galdames-Retamal J. (2021). On IoT-Friendly Skewness Monitoring for Skewness-Aware Online Edge Learning. Appl. Sci..

[21] Banos O., Galvez J.-M., Damas M., Pomares H., Rojas I. (2014). Window Size Impact in Human Activity Recognition. Sensors.

[22] Tola D., Corke P. (2023). Understanding URDF: A Dataset and Analysis. arXiv.

[23] Robotics Toolbox Peter Corke. https://petercorke.com/toolboxes/robotics-toolbox/.

[24] Siciliano B., Sciavicco L., Villani L., Oriolo G. (2009). Kinematics. Robotics: Modelling, Planning and Control.

[25] Mann K.A., Wernere F.W., Palmer A.K. (1989). Frequency spectrum analysis of wrist motion for activities of daily living. J. Orthop. Res..

[26] Green J.M. (1995). Examination of Control and Co-Ordination of Fast Versus Slow Movements in Patients Following Stroke. Ph.D. Thesis.

[27] Hingtgen B., McGuire J.R., Wang M., Harris G.F. (2006). An upper extremity kinematic model for evaluation of hemiparetic stroke. J. Biomech..

[28] Ludwig S.A., Burnham K.D. (2018). Comparison of Euler Estimate using Extended Kalman Filter, Madgwick and Mahony on Quadcopter Flight Data. Proceedings of the 2018 International Conference on Unmanned Aircraft Systems (ICUAS).

[29] Bai J., Ng S. (2005). Tests for Skewness, Kurtosis, and Normality for Time Series Data. J. Bus. Econ. Stat..

[30] Blanca M.J., Arnau J., López-Montiel D., Bono R., Bendayan R. (2013). Skewness and Kurtosis in Real Data Samples. Methodology.

[31] Bernardeau F. (1994). Skewness and Kurtosis in Large-Scale Cosmic Fields. Astrophys. J..

[32] Co-Kurtosis and Capital Asset Pricing-Fang-1997-Financial Review-Wiley Online Library. https://onlinelibrary.wiley.com/doi/abs/10.1111/j.1540-6288.1997.tb00426.x.

[33] Hui E.S., Cheung M.M., Qi L., Wu E.X. (2008). Towards better MR characterization of neural tissues using directional diffusion kurtosis analysis. NeuroImage.

[34] Hui E.S., Fieremans E., Jensen J.H., Tabesh A., Feng W., Bonilha L., Spampinato M.V., Adams R., Helpern J.A. (2012). Stroke Assessment with Diffusional Kurtosis Imaging. Stroke.

[35] Rosenkrantz A.B., Padhani A.R., Chenevert T.L., Koh D., De Keyzer F., Taouli B., Le Bihan D. (2015). Body diffusion kurtosis imaging: Basic principles, applications, and considerations for clinical practice. J. Magn. Reson. Imaging.

